# *Ganoderma lucidum* alcohol extract targets mutant *p53* signaling to inhibit proliferation in triple-negative breast brain metastasis cancer

**DOI:** 10.7150/jca.133558

**Published:** 2026-05-11

**Authors:** Yi-Chung Chien, Guo-Wei Wu, Yu-Heng Lin, Jia-Yan Wu, Yung-Luen Yu

**Affiliations:** 1 Department of Physiology, School of Medicine, China Medical University, Taichung 406040, Taiwan.; 2 Center for Molecular Medicine, China Medical University Hospital, Taichung 404327, Taiwan.; 3 Graduate Institute of Biomedical Sciences, China Medical University, Taichung 406040, Taiwan.; 4 Institute of Translational Medicine and New Drug Development, China Medical University, Taichung 406040, Taiwan.; 5 Office of Research and Development, Asia University, Taichung 413305, Taiwan.

**Keywords:** triple-negative breast cancer, brain metastasis, p53, *Ganoderma lucidum*

## Abstract

Triple-negative breast cancer (TNBC) is among the most aggressive subtypes of breast cancer, frequently characterized by invasive growth, metastasis, and poor clinical outcomes. Brain metastasis, commonly observed in TNBC patients, significantly worsens prognosis and survival rates. Current therapeutic strategies for TNBC brain metastases remain limited and largely ineffective. Mutant p53, common in TNBC, promotes malignancy and metastasis. MDA-MB-231 cells and their brain metastatic variant (MDA-MB-231-Br) harbor a p53 R280K mutation. *Ganoderma lucidum*, historically known for anticancer properties, has unclear effects on breast cancer brain metastasis. This study evaluated the impact of alcohol-extracted *Ganoderma lucidum* on TNBC brain metastasis cells via mutant p53 inhibition. Treatment with 10% *G. lucidum* alcohol extract for 48 hours significantly reduced proliferation in MDA-MB-231 and MDA-MB-231-Br cells. Knockdown of p53 confirmed its role in cell proliferation. Additionally, MG132, proteasome inhibitor, restored p53 levels, indicating *G. lucidum* extract promotes p53 degradation. Collectively, our findings suggest that alcohol-extracted *G. lucidum* inhibits the proliferation of TNBC brain metastatic cells by promoting the degradation of mutant p53. This mechanistic insight provides a potential therapeutic strategy for mitigating brain metastasis in triple-negative breast cancer.

## Introduction

Triple-negative breast cancer (TNBC) is an aggressive breast cancer subtype defined by the lack of estrogen receptor, progesterone receptor, and HER2 expression 1. It accounts for roughly 10-15% of all breast cancers yet carries the poorest prognosis among breast cancer subtypes 2. Clinically, TNBC is characterized by early visceral metastasis and high relapse rates compared to other breast cancer subtypes 2, 3. Notably, the central nervous system is a common site of metastatic spread: approximately one-third to one-half of patients with advanced TNBC will develop brain metastases over the course of their disease. These breast cancer brain metastases (BCBM) portend dismal outcomes, with limited therapeutic options and a median survival often measured in months. The propensity of TNBC to colonize the brain is linked to both intrinsic tumor aggressiveness and unique interactions within the brain microenvironment. Metastatic TNBC cells must cross the blood-brain barrier and adapt to the neural niche, where they engage in complex “seed and soil” interactions with resident cells. Astrocytes and microglia in the metastatic niche secrete pro-inflammatory cytokines and other factors that can paradoxically support tumor cell survival and outgrowth 4. For instance, activation of astrocyte inflammasomes and resultant interleukin-1β release have been shown to fuel TNBC metastasis in the brain 4. The pro-inflammatory, tumor-promoting milieu in the brain represents a major challenge in treatment. Because effective targeted therapies for TNBC brain metastases are lacking, these tumors remain a therapeutic dead-end and an area of urgent clinical need 2, 4. This context underscores the importance of identifying novel therapeutic approaches that can both inhibit TNBC tumor cell growth and modulate the prometastatic brain microenvironment 5.

One promising molecular target in TNBC is the p53 tumor suppressor pathway. The TP53 gene, which encodes the p53 protein, is mutated in the majority of TNBC cases which is over 80% of TNBC tumors harbor p53 mutations 6. Wild-type p53 normally safeguards genomic integrity by inducing cell cycle arrest or apoptosis in response to cellular stress. In TNBC, however, p53 is frequently inactivated by missense mutations that not only abrogate its tumor-suppressive functions but also often endow the mutant protein with oncogenic gain-of-function properties 7. Mutant p53 proteins can drive tumor progression by promoting uncontrolled proliferation, invasion, metastasis, and resistance to therapy 7. Consistent with these effects, TP53 mutations are associated with more aggressive disease and poorer patient outcomes in breast cancer 8. Importantly, p53 aberrations appear to play a role in the brain metastatic process. Studies have found that TP53 mutation is the most common genetic alteration in breast cancer brain metastases, and p53 pathway dysfunction is enriched in brain-seeking TNBC cells. These observations suggest that loss of p53's tumor suppressor activity (and/or gain of pro-metastatic functions) facilitates the colonization of the brain 9. Consequently, there is strong rationale to therapeutically target mutant p53 in TNBC. Indeed, the prevalence of TP53 mutations in TNBC has sparked interest in it as a targetable vulnerability 10. Early-stage clinical efforts have explored small molecules that restore wild-type p53 activity or destabilize mutant p53, with some compounds (e.g. APR-246) showing selective efficacy in mutant p53-expressing TNBC cells 10. By aiming at this high-frequency driver mutation, such approaches seek to suppress TNBC growth and potentially impair its metastatic spread. Therefore, mutant p53 is a central contributor to TNBC pathogenesis and metastasis, and strategies to counteract mutant p53 could be especially impactful in combating aggressive manifestations like brain metastases.

*Ganoderma lucidum* (Reishi or Lingzhi), a medicinal mushroom used for centuries in East Asia, has emerged as a potential source of anti-cancer and anti-inflammatory agents. Alcohol extracts of *G. lucidum* (which are enriched in triterpenoids) and aqueous extracts (rich in polysaccharides) have been investigated in various disease contexts. A broad body of research documents the intrinsic immunomodulatory and anti-tumor properties of *G. lucidum* 11. *In vitro* and *in vivo* studies have shown that *G. lucidum* extracts can interfere with cell cycle progression, induce apoptosis, and suppress angiogenesis in cancer models 11. For example, alcohol-extracted *G. lucidum* significantly reduced the viability and migratory capacity of TNBC cells (MDA-MB-231) in culture, while also downregulating pro-inflammatory cytokines (IL-6, IL-8) and matrix metalloproteinases (MMP-2, MMP-9) that facilitate invasion 12. These findings indicate that *G. lucidum* can exert direct anti-proliferative and anti-metastatic effects on tumor cells. In addition, *G. lucidum* demonstrates potent anti-inflammatory activity, which may mitigate the supportive tumor microenvironment 13. Notably, an ethanol extract of *G. lucidum* was shown to suppress microglial activation by inhibiting NF-κB and Toll-like receptor 4 signaling, thereby lowering the production of inflammatory mediators (NO, PGE2, TNF-α, IL-1β) in LPS-stimulated brain microglial cells 11. This dual ability to attack tumor cells and dampen inflammation is particularly relevant in the context of TNBC brain metastases, where cancer cell invasiveness and neuroinflammation go hand in hand. At the molecular level, *G. lucidum* contains bioactive triterpenes (e.g. ganoderic acids) and polysaccharides that modulate key signaling pathways in cancer. Intriguingly, recent evidence suggests that *G. lucidum* polysaccharides can restore mutant p53's tumor-suppressive function in cancer cells. Therefore, treating mutant p53-expressing tumor cells with *G. lucidum* led to reactivation of p53 target genes for growth inhibition, and apoptosis 14. This observation raises the possibility that components of *G. lucidum* may directly target the mutant p53 pathway. Taken together, current research supports the concept that alcohol-extracted *G. lucidum* has multifaceted anti-cancer effects, including pro-apoptotic, anti-invasive, and anti-inflammatory actions. Therefore, we hypothesize that alcohol-extracted *G. lucidum* could be a novel therapeutic approach to TNBC brain metastasis by simultaneously targeting tumor-intrinsic drivers (such as mutant p53) and the pro-tumor inflammatory microenvironment. The present study was designed to investigate the effects of alcohol-extracted *G. lucidum* on mutant p53-driven TNBC brain metastases, with the goal of establishing a foundation for a new integrative treatment strategy for this deadly condition.

## Materials and Methods

### Comprehensive analyses of *Tp53* from The Cancer Genome Atlas (TCGA)

The UALCAN web platform (http://ualcan.path.uab.edu/index.html) provides a user-friendly, interactive interface for exploring cancer transcriptomic and clinical datasets. This platform utilizes TCGA level-3 RNA sequencing data along with corresponding clinical information, encompassing 31 different tumor types 15. In the present study, we employed UALCAN to examine TP53 expression differences between tumor and normal tissue samples, and to evaluate the gene's prognostic significance by analyzing overall survival data in breast cancer patients. All bioinformatic analyses were performed via UALCAN, which is recognized for its validated and reproducible methodologies in cancer research 15.

### Preparation of *Ganoderma lucidum* ethanolic extract

*Ganoderma lucidum* (G. lucidum) fruiting bodies were obtained from Sancai Lingzhi Ecological Farm (New Taipei City, Taiwan) and ground into a fine powder. For extraction, 1 g of powdered material was dissolved in 10 mL of 95% ethanol and incubated in a water bath at 37 °C for 30 min. The mixture was then centrifuged at 1,000 rpm for 5 min, and the supernatant was collected. The extract was sequentially filtered through filter paper and a 0.22 μm membrane filter to remove particulate matter. The filtrate was aliquoted (1 mL per microcentrifuge tube) and concentrated using a centrifugal concentrator at room temperature for 2 h 50 min, yielding approximately 20 μL of concentrated ethanolic extract. The concentrate was reconstituted in 1 mL of serum-free culture medium, followed by centrifugation at 12,000 × g for 5 min to remove insoluble debris. The resulting supernatant was collected as the final G. lucidum extract, with a concentration of 15 mg/mL. For experimental treatments, the stock solution (15 mg/mL) was defined as 100%, and working concentrations were prepared by dilution in serum-free medium (e.g., 10% corresponds to 1.5 mg/mL).

### Cell culture and treatment

Human triple-negative breast cancer cell lines MDA-MB-231 and BT549 were obtained from the American Type Culture Collection (ATCC). The TNBC brain metastasis cell line MDA-MB-231-Br was generously provided by Dr. Dihua Yu from the University of Texas MD Anderson Cancer Center 16-18. MDA-MB-231 and MDA-MB-231-Br cells were maintained in DMEM/F12 medium supplemented with 10% fetal bovine serum (FBS; Gibco™) and 1% penicillin-streptomycin (Gibco™), while BT549 cells were cultured in RPMI-1640 medium supplemented with the same components. The p53 point mutations present in these cell lines were R280K in MDA-MB-231 and MDA-MB-231-Br cells, and R249S in BT549 cells, as previously reported 19, 20. All cells were incubated at 37 °C in a humidified atmosphere containing 5% CO₂. For treatment, cells were exposed to 10% G. lucidum extract (alcohol-extracted *Ganoderma lucidum*; equivalent to 1.5 mg/mL) and/or the proteasome inhibitor MG132 (HY-13259, MedChemExpress), with a stock concentration of 3 mM and a final working concentration of 3 μM.

### IncuCyte ZOOM live-cell imaging and cytotoxicity assay

MDA-MB-231 and MDA-MB-231-Br cells (3 × 10³ cells/well) were seeded in 96-well plates and allowed to adhere overnight. Cells were then treated with different concentrations of alcohol-extracted *Ganoderma lucidum* (0%, 2.5%, 5%, and 10%) and imaged using the IncuCyte ZOOM Live-Cell Analysis System (Essen BioScience). Time-lapse imaging was performed under 37 °C and 5% CO₂ conditions for 24-72 h at predefined intervals. Quantitative analysis of cell proliferation and cytotoxicity was performed using the IncuCyte ZOOM software. Each experiment was independently repeated at least three times.

### Cell viability assay

Cell viability was assessed using a WST-1 assay (Abcam, Cambridge, UK). This assay is based on the ability of viable cells to convert the water-soluble yellow tetrazolium salt WST-1 into an orange-red formazan dye via mitochondrial dehydrogenase activity. Cells were seeded in 96-well plates at an initial density of 3,000 cells per well and incubated for 24 h. Subsequently, cells were treated with G. lucidum for 48 h. After treatment, WST-1 reagent was diluted 1:10 in serum-free culture medium and added to each well. The plates were incubated at 37°C for 0.5-2 h. Absorbance was measured at 450 nm using a microplate reader (BioTek Synergy 2). Cell viability was calculated based on the OD values.

### Western blot analysis

Cells were lysed, and the lysates were centrifuged at 12,000 × g for 15 min at 4 °C to collect the supernatant. Protein concentrations were determined by measuring absorbance at 595 nm using a microplate reader. Equal amounts of protein were denatured at 99 °C for 5 min, separated by 10-12% SDS-PAGE, and subsequently transferred onto polyvinylidene difluoride (PVDF) membranes. The membranes were blocked with 5% non-fat dry milk in Tris-buffered saline containing 0.1% Tween-20 (TBST) for 1 h at room temperature, followed by incubation with primary antibodies overnight at 4 °C. After three washes with TBST, membranes were incubated with horseradish peroxidase (HRP)-conjugated secondary antibodies for 1 h at room temperature. Protein bands were detected using an enhanced chemiluminescence (ECL) detection system (Millipore, Billerica, MA, USA) and visualized with a ChemiDoc™ imaging system (Bio-Rad, Hercules, CA, USA). Band intensities were quantified using ImageJ software (National Institutes of Health, Bethesda, MD, USA), with β-actin used as a loading control. The following primary antibodies were used: anti-p53 (Santa Cruz Biotechnology, sc-126; 1:500), anti-α-tubulin (GeneTex, GTX112141; 1:2000), and anti-β-actin (GeneTex, GTX109639; 1:5000).

### Gene knockdown by shRNA

Gene knockdown was performed using lentiviral short hairpin RNA (shRNA) constructs targeting *TP53* (TRCN0000010814; target sequence: GAGGGATGTTTGGGAGATGTA). A corresponding empty vector (ASN0000000001) was used as a control. All constructs were obtained from the RNAi Core Facility. Plasmid DNA was amplified in bacteria and purified following 8-16 h of culture. HEK293T cells were seeded in 6-well plates and transfected at 60-70% confluency using Lipofectamine™ (Invitrogen, Carlsbad, CA, USA) to generate lentiviral particles. Viral supernatants were subsequently collected and used to infect target cells. At 48 h post-infection, cells were subjected to puromycin selection for 3-5 days to establish stable knockdown cell lines. Knockdown efficiency was validated by quantitative real-time PCR (qRT-PCR) and/or Western blot analysis. For qRT-PCR, the following primers were used: human *TP53* forward, 5'-GCGCACAGAGGAAGAGAATC-3'; human *TP53* reverse, 5'-CTCTCGGAACATCTCGAAGC-3'; human *GAPDH* forward, 5'-CCACCCATGGCAAATTCCATGGCA-3'; and human *GAPDH* reverse, 5'-TCTAGACGGCAGGTCAGGTCCACC-3'.

### Statistical analysis

All data are presented as mean ± standard deviation (SD) from at least three independent experiments. Statistical significance between control and treatment groups was determined using a two-tailed Student's t-test. P < 0.05 (*), P < 0.01 (**), and P < 0.001 (***) were considered statistically significant, while n.s. denotes no significant difference.

## Results

### Expression levels of *TP53* (*p53*) in clinical specimens and its impact on survival rates

**Figure 1:**
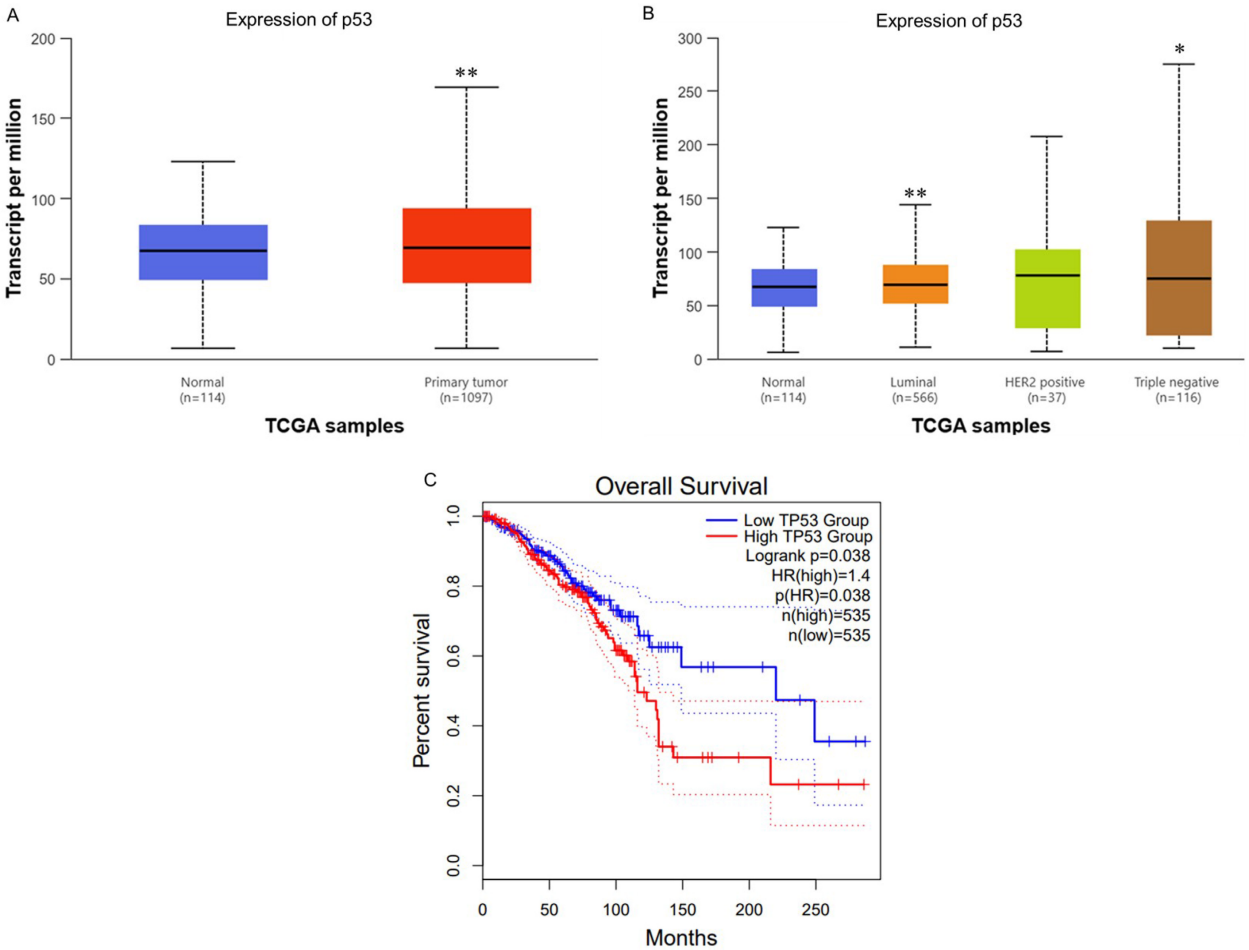
** Expression Levels of TP53 (p53) in Clinical Specimens and Its Impact on Survival Rates (A)** TP53 (p53) mRNA levels are significantly higher in breast tumor tissue than in normal breast tissue (p=6.20×10^-4^). **(B)** Stratification by molecular subtype shows luminal and triple-negative (TNBC) tumors with elevated TP53 expression compared to normal (p=6.56×10^-3^ and 1.61×10^-2^, respectively), whereas HER2-positive tumors do not differ significantly. **(C)** Kaplan-Meier analysis reveals that high TP53 expression is associated with significantly shorter overall survival (log-rank p<0.05).

To ascertain the role and significance of *p53* in breast cancer (BC), we utilized UALCAN for analyzing BC patient data from The Cancer Genome Atlas (TCGA) database. We aimed to compare *p53* expression between normal and cancerous tissues, different subtypes, and evaluate its impact on patient survival. First, we analyzed *TP53* RNA expression levels across normal and BC tissues. The results indicated significantly higher *p53* expression in BC tissues compared to normal tissues (Fig. 1A). Second, we also evaluated TP53 levels among different subtypes BC. The results indicated that Luminal and TNBC subtypes are significantly higher than normal tissues and HER2 positive subtypes (Fig. 1B). This results further supporting its role in cancer progression. Finally, the Kaplan-Meier survival analysis demonstrated a significant correlation between *TP53* expression and overall survival (OS). Patients with higher *TP53* expression showed notably shorter OS compared to those with lower expression levels (Fig. 1C). This suggests that elevated *TP53* expression is associated with poorer prognosis in BC patients. Therefore, our findings underscore the potential significance of TP53 as a biomarker for breast cancer progression and prognosis.

### Expression levels of wild type and mutant *TP53* (*p53*) in clinical specimens and its impact on survival rates

**Figure 2:**
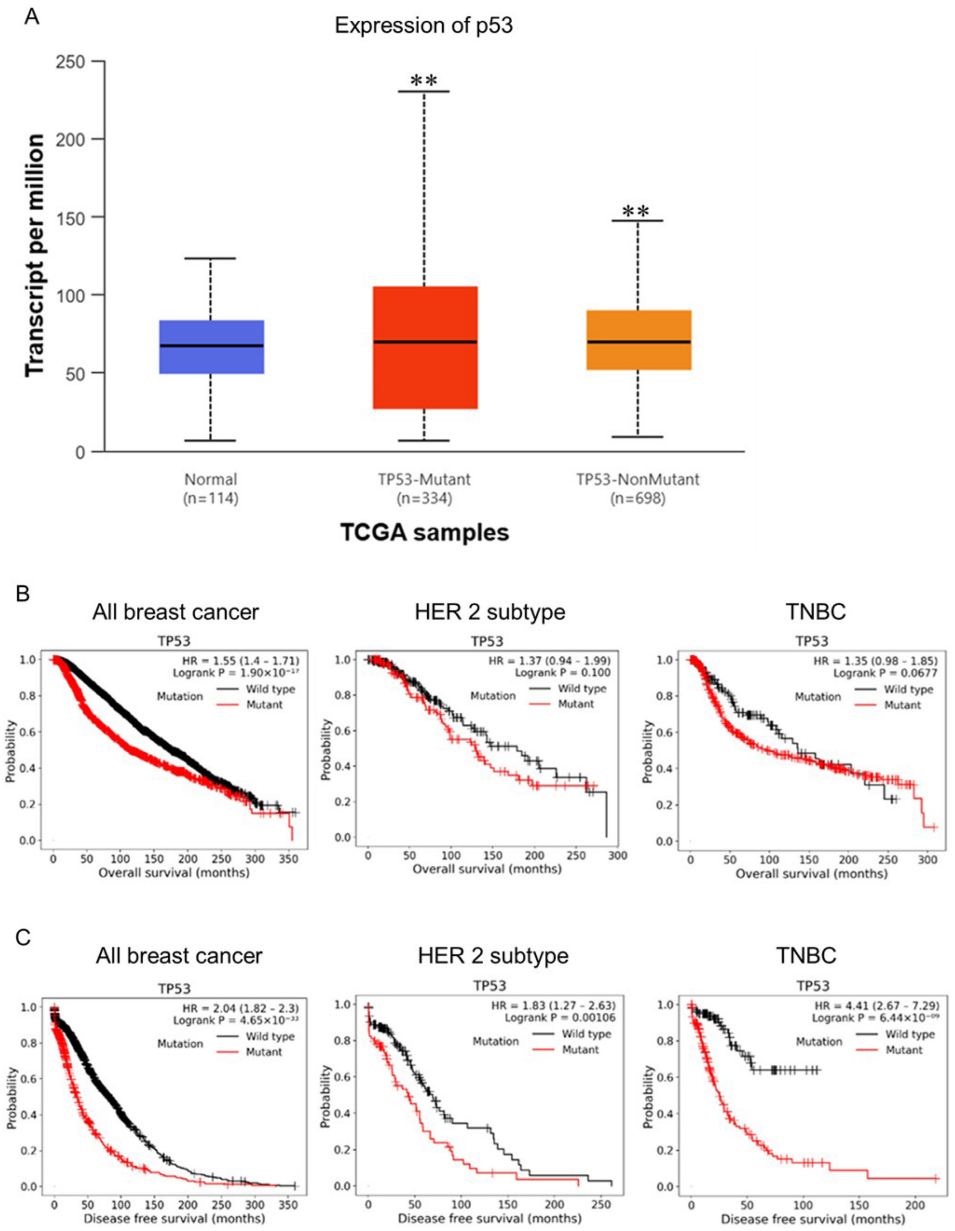
** Expression Levels of Wild Type and Mutant TP53 (p53) in Clinical Specimens and Its Impact on Survival Rates. (A)** TCGA/UALCAN analysis of TP53 mutation frequency in normal versus breast cancer tissues. **(B)** Kaplan-Meier overall survival (OS) curves stratified by TP53 mutation status in total breast cancer, HER2+, and TNBC subtypes; TP53 mutations are associated with significantly worse OS in total breast cancer (HR = 1.55; p = 1.9×10⁻¹⁷) but not in HER2+ or TNBC (p = 0.1 and 0.067, respectively). **(C)** Kaplan-Meier disease-free survival (DFS) curves by TP53 mutation status, showing that TP53 mutations correlate with inferior DFS in total breast cancer (HR = 2.04; p = 4.65×10⁻³³) and within the HER2+ (HR = 1.83; p = 0.00106) and TNBC (HR = 4.41; p = 6.44×10⁻⁹) subtypes.

p53 is a sequential transcription factor encoded by the *TP53* gene and is known as the "guardian of the genome" because it senses DNA damage in cells and turns on its downstream signaling to induce DNA repair, cell cycle arrest and apoptosis by regulating the expression of its target genes. Once the p53 pathway is altered, genetically damaged cells will not enter a state of senescence or apoptosis, leading to the accumulation of mutations and the acquisition of cancer markers. Surprisingly, p53 is mutated in over half of all human cancers 21. Therefore, we further analysis the wild type and mutant p53 in BC. We found that the ratio between p53-mutant and p53-nonmutant is about 1:2 and both significantly higher in BC tissues compared to normal tissues (Fig. 2A). This result might indicated that the mutant p53 plays a role in the progression of BC. Next, we evaluated the correlation OS between wild type and mutant p53 in all breast cancer, HER2 positive, and TNBC subtypes. As the results shown, we found mutant p53 cause poor survival compared to wild type p53 in all breast cancer (HR=1.55, p=1.9x10^-17^). While the HER2 positive and TNBC subtypes reveal non-significant different between wild type and mutant p53 (p=0.1 and p=0.067) (Fig. 2B). In addition to overall survival, we interrogated disease-free survival (DFS) according to TP53 mutation status across breast cancer cohorts. TP53-mutant tumors were consistently associated with inferior DFS compared with TP53-wild-type tumors in the overall breast cancer population (HR = 2.04, 95% CI 1.82-2.30; log-rank P = 4.65 × 10⁻³³). This adverse DFS association remained significant in the HER2 subtype (HR = 1.83, 95% CI 1.27-2.63; log-rank P = 0.00106) and was particularly pronounced in TNBC (HR = 4.41, 95% CI 2.67-7.29; log-rank P = 6.44 × 10⁻⁹), indicating that TP53 mutation is strongly linked to earlier disease events, with the largest effect size observed in TNBC (Fig. 2C). Collectively, our findings suggest that mutant p53 may be a key contributor to TNBC progression. These data implicate mutant p53 as a potential driver of TNBC progression. Our results indicate that mutant p53 could represent an important prognostic or therapeutic factor in TNBC.

### Alcohol-extracted *Ganoderma lucidum* modulates p53 protein in TP53-mutant TNBC models

**Figure 3:**
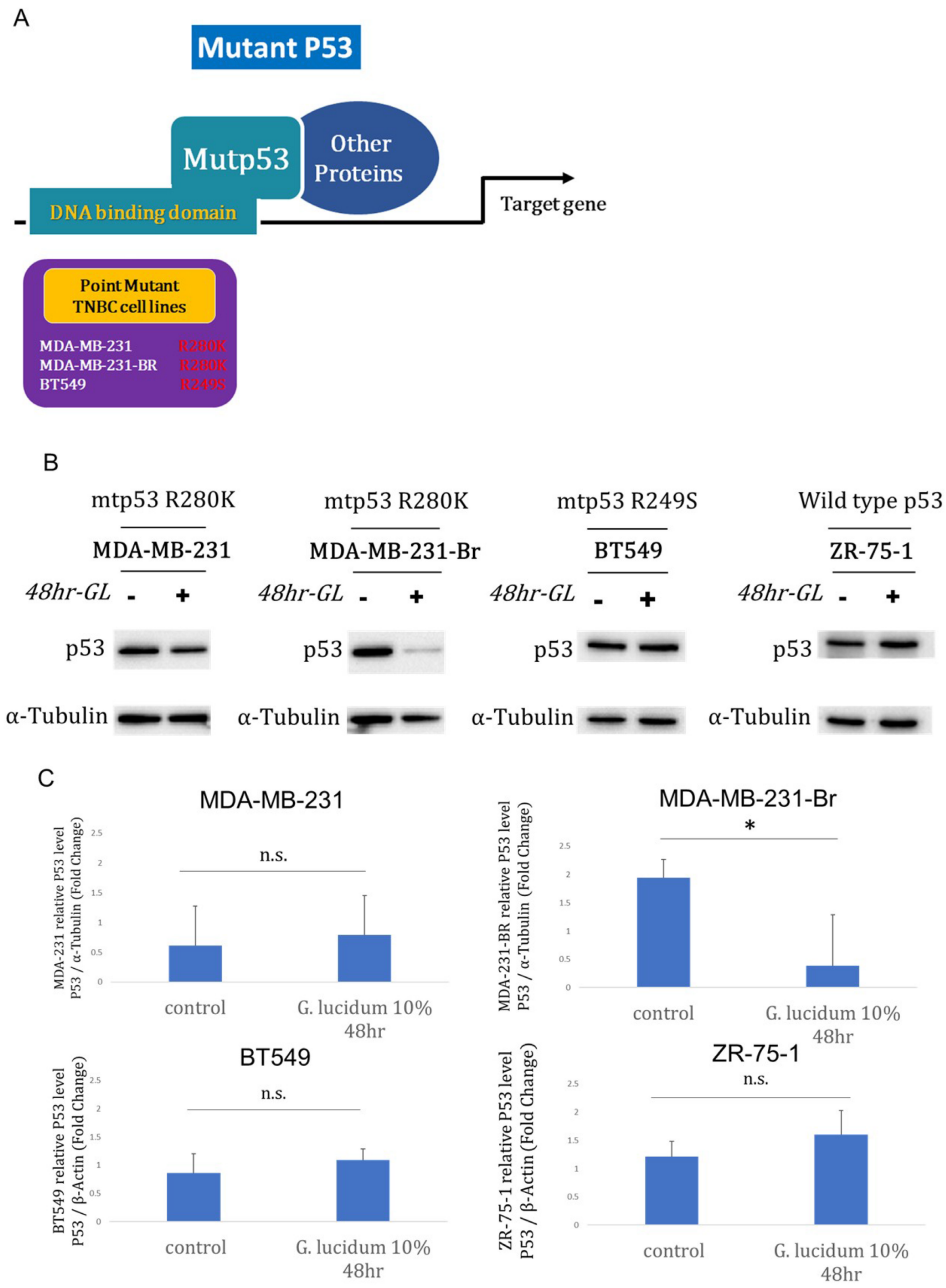
** Alcohol-extracted *Ganoderma lucidum* selectively downregulates mutant p53 protein in TNBC cell models. (A)** Schematic of the TP53-mutant TNBC cell lines used: MDA-MB-231 (R280K), BT549 (R249S), and the brain metastatic derivative MDA-MB-231-Br (R280K). **(B)** Western blot analysis of p53 protein following treatment with 10% alcohol-extracted *Ganoderma lucidum* for 48 hours shows marked downregulation of p53 in MDA-MB-231-Br, with minimal effect in MDA-MB-231 and BT549. **(C)** Quantification of p53 protein levels normalized to β-actin confirms a significant reduction only in MDA-MB-231-Br cells, indicating context-specific susceptibility of mutant p53 to extract-mediated degradation.

It has been found that mutant p53 is the most commonly mutated gene in TNBC and TNBC brain metastasis. But the molecular mechanism is still unknown. Therefore, we have prepared three p53 mutant TNBC cell lines including two mutants (R280K and R249S) for following experiments (Fig. 3A). Next, we profiled mutant p53 protein responses to alcohol-extracted *Ganoderma lucidum* (10%, 48 h) across TP53-mutant TNBC models harboring R280K or R249S. In western blots, p53 abundance was markedly reduced in MDA-MB-231-Br after treatment, whereas MDA-MB-231 and BT549 showed minimal visible change (Fig. 3B and 3C). Together, the data indicate context-dependent suppression of mutant p53, most pronounced in the brain-derived MDA-MB-231-Br variant, consistent with selective vulnerability in metastatic settings.

### Proteasome-linked between alcohol-extracted *Ganoderma lucidum* and p53 degradation

**Figure 4:**
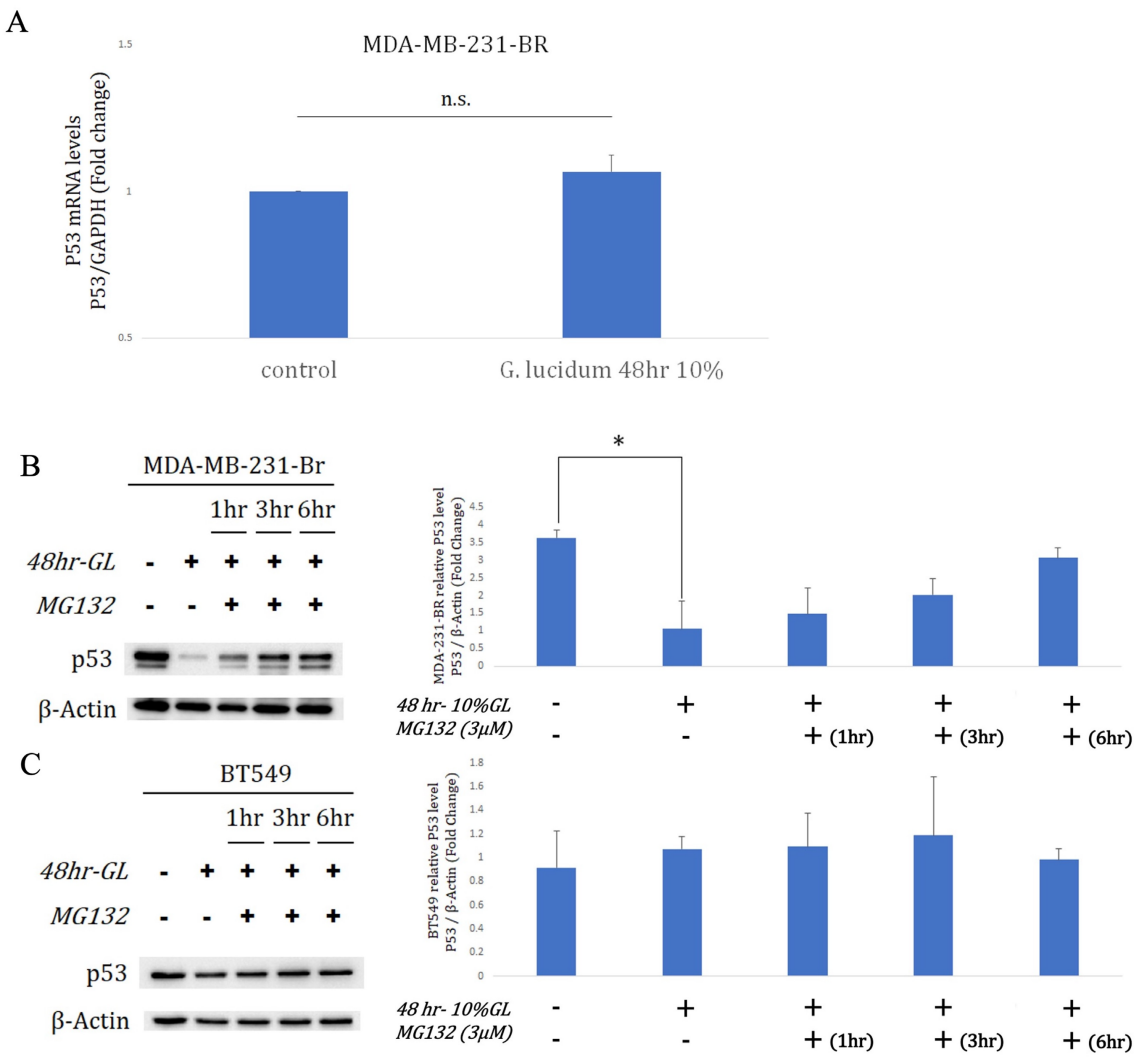
** Alcohol-extracted *Ganoderma lucidum* induces proteasome-mediated degradation of mutant p53 in a context-dependent manner. (A)** qRT-PCR analysis shows that TP53 mRNA expression remains unchanged in MDA-MB-231-Br cells after treatment with 10% alcohol-extracted *Ganoderma lucidum* for 48 hours. **(B)** Western blot analysis reveals a marked reduction in p53 protein following extract treatment, with partial restoration upon subsequent MG-132 (proteasome inhibitor) exposure for 1-6 hours, indicating proteasome-dependent degradation. **(C)** In BT549 cells harboring the TP53 R249S mutation, p53 protein levels remain unchanged following extract treatment, and MG-132 co-treatment does not rescue p53 expression. These results suggest that Ganoderma-induced mutant p53 degradation is dependent on proteasomal pathways and is context- and mutation-specific.

qRT-PCR indicated TP53/GAPDH expression remained unchanged in MDA-MB-231-Br after 10% alcohol-extracted G. lucidum for 48 h (Fig.4A). Despite stable mRNA, western blots showed a significant drop in mutant p53 protein after extract exposure, with β-actin as loading control (Fig.4B). MG-132 (concentration unspecified) administered after the 48-h extract treatment partially restored p53 levels over 1-6 h, supporting proteasome-mediated degradation in MDA-MB-231-Br (Fig.4B). In BT549 cells harboring TP53 R249S, the same extract regimen (10%, 48 h) did not lower p53 protein relative to β-actin (Fig.4C). Subsequent MG-132 addition for 1, 3, or 6 h produced no consistent restoration, indicating limited proteasome involvement in BT549 under these conditions (Fig.4C). Together with the MG-132 reversal in MDA-MB-231-Br, these results support extract-triggered, proteasome-mediated p53 degradation that is context dependent. This selectivity may reflect mutation- or metastasis-associated regulation of p53 turnover, highlighting differential vulnerability across TP53-mutant TNBC models. Overall, alcohol-extracted G. lucidum promotes proteasome-dependent mutant p53 loss in MDA-MB-231-Br, but not in BT549.

### Mutant p53 supports MDA-MB-231Br growth

**Figure 5:**
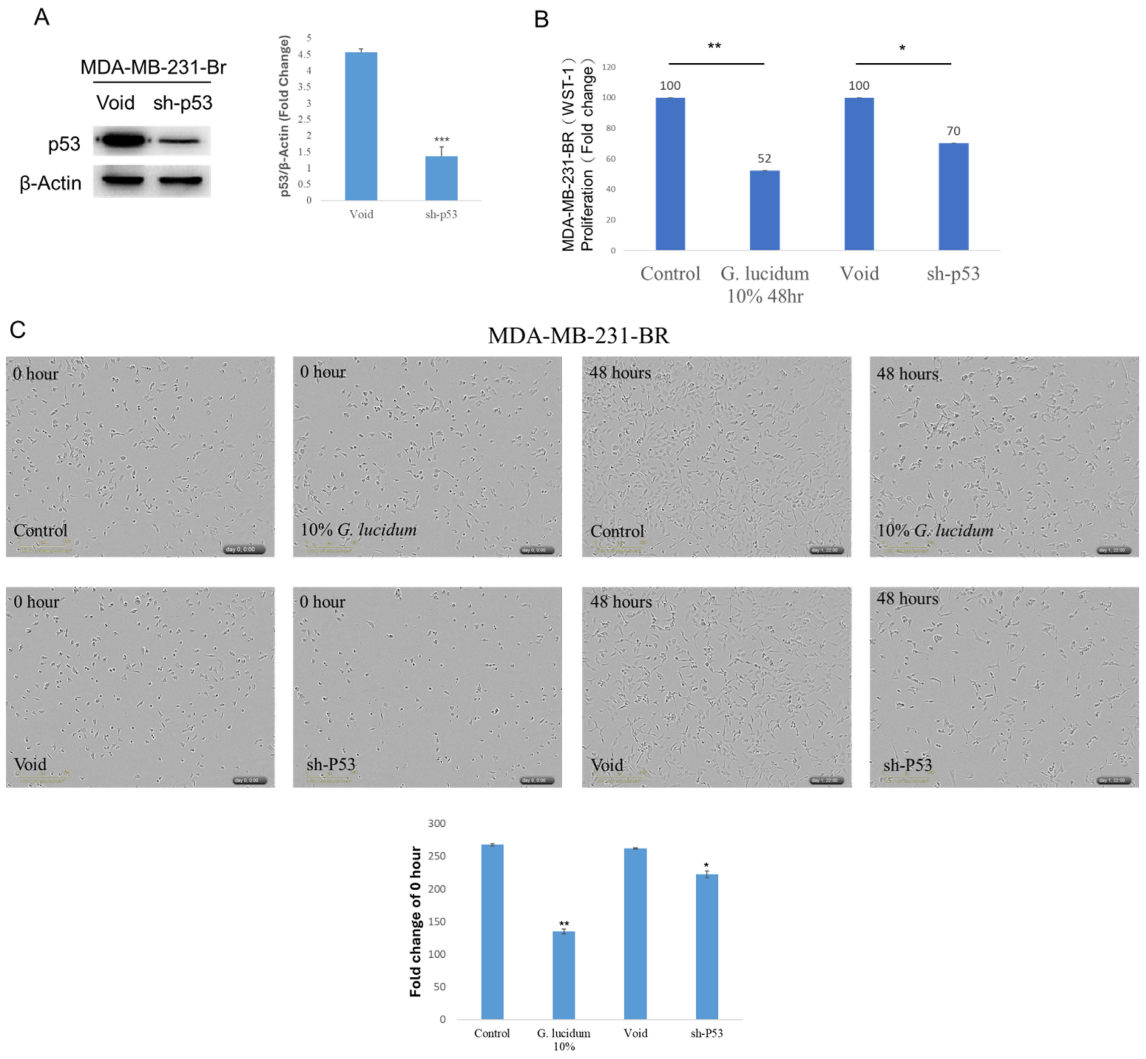
** Mutant p53 promotes proliferation of brain-metastatic TNBC cells and is functionally suppressed by alcohol-extracted *Ganoderma lucidum*. (A)** Western blot analysis confirms effective knockdown of p53 protein in MDA-MB-231-Br cells transduced with TP53-targeting shRNA compared to control (void), normalized to β-actin. **(B)** WST-1 cell viability assays show that p53 silencing decreases proliferation to ~70% of control levels, while alcohol-extracted *Ganoderma lucidum* treatment (10%, 48 h) further suppresses proliferation to ~52%. These results suggest that mutant p53 drives cell growth in MDA-MB-231-Br cells and that alcohol-extracted *Ganoderma lucidum* phenocopies its loss. **(C)** The IncuCyte ZOOM live-cell imaging assay reveals reduced cell density in both p53-knockdown and alcohol extracted *Ganoderma lucidum* treated cells, indicating impaired proliferative capacity.

To define mutant p53 function in brain-metastatic MDA-MB-231-Br cells, TP53 was silenced using a p53-targeting shRNA. p53 protein was reduced in sh-p53 cells versus void controls after β-actin normalization (Fig.5A). In WST-1 assays, sh-p53 decreased proliferation to 70% of the void group (Fig.5B). Alcohol-extracted *Ganoderma lucidum* treatment (10%, 48 h) lowered WST-1 proliferation to 52% of control (Fig.5B). We next employed the IncuCyte ZOOM live-cell imaging assay to evaluate whether treatment with alcohol-extracted *Ganoderma lucidum* and p53 knockdown could significantly inhibit the growth of MDA-MB-231-Br cells. As shown in Fig. 5C, a marked reduction in cell growth was observed in both groups after 48 hours. Among them, the alcohol-extracted *Ganoderma lucidum* treatment group exhibited a more pronounced inhibitory effect. Together, these findings support a pro-proliferative role for mutant p53 in MDA-MB-231-Br, and suggest Alcohol-extracted *Ganoderma lucidum* broadly phenocopies mutant p53 depletion.

## Discussion

TNBC remains one of the most clinically aggressive and therapeutically challenging subtypes of breast cancer, especially when metastasis to the brain occurs. Brain metastases are frequently observed in patients with advanced TNBC and are associated with severely limited treatment options and poor prognosis 1. A central molecular contributor to TNBC progression is mutant p53, which is mutated in over 80% of TNBC cases and exhibits oncogenic gain-of-function (GOF) properties, including enhanced proliferation, invasion, and resistance to apoptosis 2.

In this study, we demonstrate that alcohol-extracted *Ganoderma lucidum* selectively reduces mutant p53 protein levels in MDA-MB-231-Br brain metastatic TNBC cells without affecting TP53 mRNA expression. Restoration of p53 protein upon MG-132 co-treatment indicates that alcohol-extracted *Ganoderma lucidum* induces proteasome-dependent degradation of mutant p53. This aligns with earlier studies suggesting that certain natural compounds, including triterpenoids derived from G. lucidum, may modulate components of the ubiquitin-proteasome system (UPS). Jiang et al. previously reported that G. lucidum polysaccharides restore p53 tumor-suppressive function in colorectal cancer models via modulation of mutant p53 14.

Our findings also reveal that alcohol-extracted *Ganoderma lucidum* treatment significantly suppresses proliferation in MDA-MB-231-Br cells. The anti-proliferative effect closely mirrors that of p53 knockdown using shRNA, indicating that mutant p53 plays a functionally pro-tumorigenic role in brain metastatic TNBC cells. This is consistent with studies reporting that mutant p53 facilitates metastasis and therapeutic resistance in breast cancer brain metastases 9, 22. Notably, this degradation effect is context-dependent, as alcohol-extracted *Ganoderma lucidum* failed to reduce p53 protein in BT549 cells harboring a different p53 mutation (R249S). These results suggest that p53 allelic context or cellular proteostasis machinery may influence alcohol-extracted *Ganoderma lucidum*-mediated degradation.

From a structural perspective, the p53 R280K mutation in MDA-MB-231-Br cells is located within the DNA-binding domain and represents a contact mutation that does not significantly disrupt the overall folding of the protein 23. As a result, this mutant p53 remains amenable to regulation through the canonical MDM2-mediated ubiquitination pathway, leading to proteasomal degradation. Consistently, treatment with the proteasome inhibitor MG132 restores p53 protein levels, supporting the involvement of the ubiquitin-proteasome system. In contrast, the p53 mutation in BT549 cells is a structural mutation that disrupts proper folding and compromises the stability of the DNA-p53 complex 24. Misfolded p53 proteins are preferentially recognized by the cellular protein quality control machinery and are degraded via chaperone-associated pathways involving molecular chaperones and the E3 ubiquitin ligase CHIP 25. Based on the combined MG132 and alcohol-extracted *Ganoderma lucidum* treatment results, we propose that alcohol-extracted *Ganoderma lucidum* primarily targets the MDM2-dependent ubiquitination pathway. This may explain its efficacy in structurally preserved mutants such as R280K, while showing limited activity in structural mutants that are predominantly degraded through chaperone/CHIP-mediated mechanisms.

Beyond its effects on p53, G. lucidum extracts have been shown to attenuate inflammation and inhibit NF-κB signaling in glial cells 11, which may be particularly relevant in the neuroinflammatory microenvironment of TNBC brain metastases. These dual anti-proliferative and anti-inflammatory properties suggest that G. lucidum may offer a two-pronged therapeutic benefit.

Nevertheless, limitations remain. We only conducted preliminary assessments of the effects of the extract on mutant p53 in TNBC cell lines. The chemical composition of the extract remains undefined, the active triterpenoids have not been isolated, and all results were derived from *in vitro* models. Dose-dependent activity, pharmacokinetics, and *in vivo* effects, particularly in orthotopic or brain metastatic models, remain to be determined. Moreover, the stability and interactions between the extract and different p53 mutants (e.g., R280K versus R249S) require further investigation. Although alcohol-extracted *Ganoderma lucidum* was observed to reduce mutant p53 levels and inhibit cell proliferation, the direct regulation of p53 function and the causal relationship with proliferation inhibition remain to be clarified; future studies could employ relevant strategies to determine whether p53 activity mediates the biological effects of alcohol-extracted *Ganoderma lucidum*. The mechanism by which alcohol-extracted *Ganoderma lucidum* promotes p53 degradation, including whether canonical and non-canonical downstream targets are affected, as well as the involvement of E3 ligases or chaperone complexes in regulating proliferation and invasion, also warrants further detailed investigation.

## Conclusion

In summary, our findings demonstrate that alcohol-extracted *Ganoderma lucidum* suppresses proliferation in brain metastatic TNBC cells by promoting proteasome-dependent degradation of mutant p53. This pharmacologic effect mimics p53 knockdown and underscores the functional importance of mutant p53 in sustaining the malignant phenotype in TNBC brain metastasis. These results highlight the potential of *G. lucidum*-derived compounds as novel modulators of mutant p53 proteostasis and suggest their utility as part of an integrative therapeutic strategy for advanced TNBC. Further studies are needed to isolate active components, define mechanistic targets, and validate *in vivo* efficacy.
